# Effectiveness of additional follow-up telephone counseling in a smoking cessation clinic in Beijing and predictors of quitting among Chinese male smokers

**DOI:** 10.1186/s12889-016-2718-5

**Published:** 2016-01-22

**Authors:** Lei Wu, Yao He, Bin Jiang, Fang Zuo, Qinghui Liu, Li Zhang, Changxi Zhou, Miao Liu, Hongyan Chen, KK Cheng, Sophia S. C. Chan, Tai Hing Lam

**Affiliations:** 1 Department of Epidemiology, Institute of Geriatrics, Chinese PLA General Hospital, 28 Fuxing Road, Beijing, 100853 China; 2 Beijing Key Laboratory of Aging and Geriatrics, Chinese PLA General Hospital, Institute of Geriatrics, 28 Fuxing Road, Beijing, 100853 China; 3 State Key Laboratory of Kidney Disease, Chinese PLA General Hospital, 28 Fuxing Road, Beijing, 100853 China; 4 Nanlou Faculty of Clinical Medicine, Department of Acupuncture, Chinese PLA General Hospital, 28 Fuxing Road, Beijing, 100853 China; 5 Nanlou Faculty of Clinical Medicine, Department of Respiration, Chinese PLA General Hospital, 28 Fuxing Road, Beijing, 100853 China; 6 Nanlou Faculty of Clinical Medicine, Department of Rehabilitation, Chinese People’s Liberation Army General Hospital, Beijing, China; 7 Public Health, Epidemiology and Biostatistics, University of Birmingham, Birmingham, UK; 8 School of Nursing, The University of Hong Kong, Hong Kong, China; 9 Department of Community Medicine and School of Public Health, The University of Hong Kong, Hong Kong, China

**Keywords:** Face-to-face counseling, Additional telephone follow-up counseling, Chinese male smoker, Quit rate, Predictors of quitting

## Abstract

**Background:**

No previous studies have investigated whether additional telephone follow-up counseling sessions after face-to-face counseling can increase quitting in China, and whether this strategy is feasible and effective for promoting smoking cessation is still unclear.

**Methods:**

A non-randomized controlled study was conducted in Beijing. We compared the quit rates of one group which received face-to-face counseling (FC) alone (one session of 40 min) to another group which received the same face-to-face counseling plus four follow-up sessions of brief telephone counseling (15–20 min each) at 1 week, 1, 3 and 6 month follow-up (FCF). No smoking cessation medication was provided. From October 2008 to August 2013, Chinese male smokers who sought treatment in a part-time regular smoking cessation clinic of a large general hospital in Beijing were invited to participate in the present study. Eligible male smokers (*n* = 547) were divided into two groups: FC (*n* = 149) and FCF (*n* = 398). Main outcomes were self-reported 7-day point prevalence and 6 month continuous quit rates at 12 month follow-up.

**Results:**

By intention to treat, at 12 month follow-up, the 7-day point prevalence and 6 month continuous quit rates of FC and FCF were 14.8 % and 26.4 %, and 10.7 % and 19.6 % respectively. The adjusted odds ratios (95 % confidence intervals) of quitting in FCF compared to FC was 2.34 (1.34–4.10) (*P* = 0.003) and 2.41 (1.28–4.52) (*P* = 0.006), respectively. Stepwise logistic regression showed that FCF, being married, unemployed and a lower Fagerström score were significant independent predictors of 6 month continuous quitting at 12 month follow-up.

**Conclusions:**

Using systematically collected data from real-world practice, our smoking cessation clinic has shown that the additional telephone follow-up counseling sessions doubled the quit rate.

**Electronic supplementary material:**

The online version of this article (doi:10.1186/s12889-016-2718-5) contains supplementary material, which is available to authorized users.

## Background

China has one-third of the world’s smokers, about 350 million in total. The 2010 Global Adult Survey reported that 52.9 % of men and 2.4 % of women were current smokers in China [1, 2]. To reduce the disease burden of tobacco worldwide, smoking cessation in China should play a critical role. However, in China Mainland, health care workers have little motivation to help smokers quit smoking [3]. Additionally, the effectiveness of existing smoking cessation interventions and smoking cessation services is still unclear [3, 4].

In western developed countries, studies which compared the effectiveness of various types of follow-up intervention (different frequency and intensity of counseling) after first counseling versus first counseling alone, have shown that the quit rates of smokers who received a “booster” (additional follow-up telephone counseling) were higher than those smokers who did not receive the “booster” [5–8]. After reviewing 22 Randomized Controlled Trials (RCTs), Pan et al. reported that the quit rate in the treatment group (with additional telephone counseling) was 64 % greater than that in the comparison group [9]. According to a 2013 Cochrane systematic review, Stead et al. reported that telephone counseling as an adjunct to brief intervention or counseling increased quit rates, compared to brief intervention or counseling alone, and the relative risk (95 % confidence internal) was 1.4 (1.2–1.7) [10]. It is worth noting that most of the trials, which were included in both of the above reviews, were performed in western, developed high-income countries, but none of the included trials were conducted in China Mainland.

Moreover, one of the major challenges of smoking cessation is the high relapse rate. Among smokers who quit initially, up to 80 % may relapse within a year [11]. By teaching coping skills and offering additional social support, the additional follow-up telephone counseling sessions might be an effective way to prevent relapse and to plan new quit attempts [5, 12]. Sheffer et al. reported that the 6-month abstinence rate in the treatment group (telephone counseling including relapse prevention) was 20.9 %, which was significantly higher than that of the control group (without relapse prevention, 10.6 %) [13].

Smoking cessation and cessation services are at an early stage of development in China Mainland. Both RCTs and observational studies about smoking cessation services are scarce. To our best knowledge, no previous studies have investigated whether additional telephone counseling sessions after face-to-face counseling can increase quitting in China, and whether this strategy is feasible and effective for promoting smoking cessation is still unclear. Although observational studies on smoking cessation interventions cannot provide the same degree of confidence for causal inference as RCTs, results from RCT often exaggerate the effect size because research subjects are carefully selected and are usually under exceptional care and attention [14]. Evaluation of real-world practice can provide more realistic effect size and can test the generalizability of RCT evidence when the interventions are implemented in different settings. When the results from a vigorous evaluation of an intervention in a new setting, such as in China, are consistent with RCT evidence elsewhere (which is predominantly from the West) and are widely disseminated, policy makers and health care professionals in similar settings are more likely to be motivated to implement the intervention.

Thus, we conducted a non-randomized controlled study in a real-world setting in one of the longest running smoking cessation clinics of Beijing, China. We aimed to compare the effect of one session face-to-face individual counseling plus follow-up telephone counseling with that of face-to-face counseling alone for Chinese male smokers in China Mainland. We also adjusted for the key potential confounders [15] so as to minimize the effect of confounding.

## Methods

### Study setting

This was a non-randomized controlled study which was based on the retrospective analysis of data collected systematically for the evaluation of the services in a smoking cessation clinic (SCC). We established a part-time SCC in the outpatient department of People’s Liberation Army General Hospital (with 3400 hospital beds and on average about 10,000 out-patients per day) in Beijing. The SCC, modeled after the Hong Kong Smoking Cessation Health Centre [16], was aimed to serve as a new platform for cessation research and evaluation in China Mainland. In 1996, 22 smoking cessation clinics had been set up in Beijing. Ours is one of the very few clinics which are still running and treating smokers regularly [17]. It started operation from 22 October 2008, and the service is still operational at the time of submission of the present paper. We provided services in 4 weekday evenings (Monday to Thursday, from 6:30 to 9 p.m.) by eligible physicians. These physicians should meet the criteria as follows: (1) had got a medical degree, (2) had more than 5 years of relevant workplace experience, and (3) had completed a smoking cessation training program and passed the examinations [16]. The target clients were smokers who volunteered to seek treatment at our clinic and paid 7 yuan (about U.S. $1) for the registration fee. No fees were charged for counseling. This study was approved by the Independent Ethics Committee of Chinese People’s Liberation Army General Hospital (S2013-066-01). Signed informed consent was obtained from all eligible participants.

### Subject recruitment and intervention

The inclusion criteria were: (1) current smokers (smoked daily for at least 6 months at the time of survey) [18], Chinese, aged 18 years or above, and (2) agreed to participate in the follow-up and signed an informed consent form. The exclusion criteria were: (1) disagreed and did not sign the informed consent form, (2) did not conform to the definition of current smoker, (3) cognitively impaired (such as those participants who could not understand and complete the questionnaire reliably), and (4) serious deafness.

All smokers received the same intervention at the first visit. Before counseling, the smoker’s smoking and related information was assessed using a baseline questionnaire through face-to-face interview lasting approximately 10 min. Then the physician provided individual face-to-face counseling based on Prochaska’s transtheoretical model [19] and the five ‘A’ (ask, advice, assess, assist and arrange) lasting at least 30 min. The physician assessed the stage of readiness in quitting smoking, strengthened clients’ motivation to quit smoking using the five ‘R’ (relevance, risks, rewards, roadblocks and repetition) approaches [20], and provided advice to overcome craving, psychological dependence and social-cultural factors associated with tobacco dependency [16]. No smoking cessation medication was provided.

After baseline intervention, smokers who visited our clinic from October 2008 to December 2010 (*n* = 254), had telephone follow-up by counselors at 1 week, 1, 3, 6 and 12 months. Our counselors were chosen from retired nurses, and they had completed a smoking cessation training program and passed the examinations. At follow-up, after assessing the smoking or quitting status, we added a “booster” (additional counseling). We asked whether the smokers or quitters had any problems. We provided problem-oriented suggestions or advice as appropriate, and also encouraged them to quit or maintain abstinence. Each follow-up lasted for about 15–20 min.

To study the effect of the “boosters”, we could not do an RCT in the present real-world service situation in the clinic as random allocation of the smokers into two groups with different follow-up interventions could create confusion in the smokers as they came for a service and did not expect to be randomized. Because of the uncertainty of whether the “booster” was effective, we stopped the additional follow-up telephone counseling for all the smokers first counseled in 2011. These smokers had the same telephone follow-up assessment by trained counselors at 1, 3, 6 and 12 months with only questions about smoking and quitting, but with no further counseling. Each follow-up lasted for about 2–3 min. These smokers constituted the group of face-to-face counseling only (FC, *n* = 149).

After 2011, we resumed the additional follow-up telephone sessions for all smokers. The smokers from January 2012 to August 2013 (*n* = 144), together will those from October 2008 to December 2010 formed the face-to-face counseling plus follow-up telephone counseling group (FCF group, total *n* = 398). The additional follow-up telephone sessions for all smokers were completed in August, 2014.

To enhance the integrity and quality of counseling, counselors were supervised during the entire project. Counselors called to contact the smokers of the two groups for at least 7 times at different days before considering them as lost to follow-up. Physicians who provided the baseline counseling were blinded to the subsequent grouping methods (with or without additional follow-up telephone sessions). The counselors who provided the additional follow-up telephone sessions could not be blinded to the grouping of the participants, but they did not know the aim of the present study, so that they could record the tobacco use status of smokers with minimal subjective bias.

### Data collection

Data collection was done at the first visit and each follow-up interview using standardized and structured questionnaires [16, 21, 22]. The following baseline characteristics data of each participant were collected: gender, age, marital status, educational level, occupation and family income; tobacco related questions included smoking history, smoking status, place of smoking, past quitting history, motivation to quit, and perceived confidence, importance and difficulties in quitting smoking (all three based on the scale of 1–100, denoting from the least to the most). All smokers had the Fagerström Test for Nicotine Dependence (FTND) and their dependence was classified as low (0–3), moderate (4–5) and severe (6–10) [23]. Exhaled carbon monoxide level was measured by trained technicians using a standard protocol and MicroCO [16]. Other questions included previous medical advice to quit, doctor diagnosed tobacco related chronic diseases and alcohol use. The follow-up questionnaires were similar to the baseline questionnaire, with deletion of redundant questions. We also added some questions on quitting as follows: “What was the date you started to stop smoking?”, “How many times have you quit smoking for more than 24 h?” and “When you quit smoking, did you have any withdrawal symptom?”

### Analysis

The data were entered (double entry) using Epidata (3.1) and analyzed using SPSS (Inc., Chicago, IL, USA) for Windows (19.0). The baseline characteristics were described using descriptive statistics. The prevalence of quitters by different baseline factors was compared with chi-square test. We used the forward stepwise logistic regression to identify the independent predictors of successful quitting and to calculate adjusted odds rations (ORs) and 95 % confidence intervals (CIs). Using intention to treat analysis (ITT), smokers who could not be contacted during follow-up were considered as non-quitters or non-reducers. Because the FCF group (28.1 %) had a much greater lost to follow-up rate than FC (18.8 %), and hence had greater percentages assumed to have had no improvement by ITT, complete case (per protocol) analysis was done by excluding those lost to follow-up as a sensitivity analysis. All P values were two-sided and <0.05 was considered as statistical significance.

The primary outcome of 7-day point prevalence quit rate was defined by not smoking any cigarettes during the past 7 days at 12 month follow-up, which was based on the United States Clinical Practice Guidelines [20]. The 6 month continuous quit rate was defined as not smoking any cigarettes during the past 6 months at 12 month follow-up. New quitters at 12 month follow-up was defined as still smoking cigarettes at 6 month follow-up, but not smoking any cigarettes for at least 7 days at 12 month follow-up. Relapse at 12 month follow-up was defined as not smoking any cigarettes at 6 month follow-up, but having smoked more than one cigarette each day for at least 7 days at 12 month follow-up. These were self-reported by the smokers without biochemical validation.

## Results

**Fig. 1:**
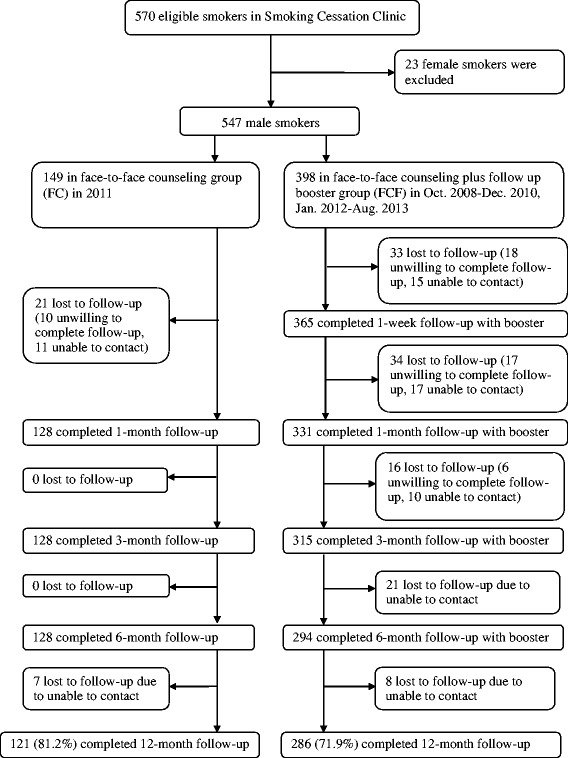
Attrition flow chart

From 22 October 2008 to 31 Aug 2013, the baseline sample included 570 eligible smokers. Because male smokers made up the majority of the smoking population in China [1], female smokers were few and had different characteristics from males, the present analysis included only 547 male smokers (149 in FC group and 398 in FCF group) and excluded 23 female smokers. Until 31 Aug 2014, 407 male smokers had completed the 12 month follow-up, 140 (25.6 %; 18.8 % in FC and 28.1 % in FCF) were lost to follow-up, mostly due to non-contacts (Fig. 1). These 140 smokers showed no differences from the 407 smokers in their baseline demographic characteristics, tobacco related and other factors (Additional file 1: Table S1).

### Demographic characteristics, tobacco related and other factors

There were no statistically significant differences between the two groups, except that the FCF group perceived less difficulty in quitting and had more willingness to pay for quitting (Table 1). Most smokers were middle aged (mean age = 41.0 years; SD = 11.3 years), married, currently employed and well-educated. More than half smoked 20 cigarettes or more each day, started smoking before 18 and had been smoking for more than 20 years. More than three-quarters had experience of quitting and had no other smokers in the household. About 40 % were severely dependent on nicotine with Fagerström score of 6–10 and were at the action stage of quitting. The mean exhaled carbon monoxide level was 12 ppm, and the mean score of perceived importance of, difficulty and confidence in quitting was 86, 73 and 68, respectively. More than half had doctor diagnosed tobacco related chronic diseases and perceived poor health status.

**Table 1:** Demographic characteristics and tobacco-related factors of 547 male smokers in two groups

	FC (*N* = 149)	FCF (*N* = 398)	*P*-value
Demographic characteristics			
Age (years) mean(SD)	41.1(10.2)	41.0(11.5)	0.89
Age (years) number (%)	N (%)	N (%)	
< 31	22 (14.8)	78 (19.6)	0.26
31–40	48 (32.2)	126 (31.7)	
41–50	54 (36.2)	114 (28.6)	
> 50	25 (16.8)	80 (20.1)	
Marital status			
Married	134 (89.9)	345 (86.7)	0.31
Single or divorced	15 (10.1)	53 (13.3)	
Education			
College and above	93 (62.4)	232 (58.3)	0.38
High school and below	56 (37.6)	166 (41.7)	
Occupation			
Currently employed	118 (79.2)	318 (79.9)	0.86
Student/unemployed/retired/others	31 (20.8)	80 (20.1)	
Family income per month (Yuan, U.S.$1 = 6 Yuan)			
< 3000	58 (38.9)	145 (36.4)	0.70
3000 ~ 6000	37 (24.8)	113 (28.4)	
> 6000	54 (36.2)	140 (35.2)	
Tobacco related factors			
Age at initiation of smoking (years)			
< 18	48 (32.2)	135 (33.9)	0.71
≥ 18	101 (67.8)	263 (66.1)	
Cigarettes smoked on average daily (cig/d)			
≥ 20	96 (64.4)	241 (60.6)	0.70
10–19	40 (26.8)	117 (29.4)	
<10	13 (8.7)	40 (10.1)	
Smoking duration (years)			
< 20	59 (39.6)	175 (44.0)	0.36
≥20	90 (60.4)	223 (56.0)	
Prior attempts to quit smoking			
0	33 (22.1)	102 (25.6)	0.40
≥ 1	116 (77.9)	296 (74.4)	
Fagerström test score			
Severe (6–10)	65 (43.6)	181 (45.5)	0.41
Moderate (4–5)	42 (28.2)	91 (22.9)	
Low (0–3)	42 (28.2)	126 (31.7)	
Exhaled CO level at first visit (mean:12 ppm)			
≥ 12	69 (46.3)	193 (48.5)	0.65
< 12	80 (53.7)	205 (51.5)	
Stage of quitting smoking			
Contemplation	43 (28.9)	91 (22.9)	0.35
Preparation	51 (34.2)	146 (36.7)	
Action	55 (36.9)	161 (40.5)	
Perceived importance of quitting (mean score:86)			
< 86	63 (42.3)	165 (41.5)	0.86
≥ 86	86 (57.7)	233 (58.5)	
Perceived difficulty in quitting (mean score:73)			
≥ 73	108 (72.5)	209 (52.5)	<0.001
< 73	41 (27.5)	189 (47.5)	
Perceived confidence in quitting (mean score:68)			
< 68	65 (43.6)	184 (46.2)	0.59
≥ 68	84 (56.4)	214 (53.8)	
Expenditure on cigarettes per day, Yuan (mean:20)			
< 20	76 (51.0)	185 (46.5)	0.35
≥ 20	73 (49.0)	213 (53.5)	
Willingness to pay for quitting, Yuan (mean:2000)			
< 2000	89 (59.7)	198 (49.7)	0.04
≥ 2000	60 (40.3)	200 (50.3)	
Perceived health status at the first visit			
Fair / poor / very poor	93 (62.4)	270 (67.8)	0.23
Very good / good	56 (37.6)	128 (32.2)	
Number of other smokers in household			
0	123 (82.6)	323 (81.2)	0.71
≥ 1	26 (17.4)	75 (18.8)	
Medical advice to quit	48 (32.2)	139 (34.9)	0.55
Had doctor diagnosed tobacco related chronic diseases	75 (50.3)	215 (54.0)	0.44
Current drinkers	106 (71.1)	274 (68.8)	0.60

### Prevalence quit rates

Table 2 and Additional file 1: Figure S1 show that by intention to treat, at 1, 3, 6 and 12 month follow-up, the 7-day point prevalence quit rate of the FC group was stable, at 16.1 %, 17.4 %, 16.1 % and 14.8 %, respectively. The rate in the FCF group rose steadily, from 18.6 % at 1 month, 23.1 % at 3 months, 25.9 % at 6 months to 26.4 % at 12 months. At 12 month follow-up, the 7-day point prevalence quit rate of the FCF group was significantly higher than that of the FC group, and the adjusted OR (95 % CI) was 2.34 (1.34–4.10), *P* = 0.003. The 6 month continuous abstinence quit rate was 10.7 % in FC and 19.6 % in FCF, and the adjusted OR (95 % CI) was 2.41 (1.28–4.52), *P* = 0.006.

**Table 2:** By intention to treat, quit rates of two groups at 1, 3, 6 and 12 month follow-up in 547 male smokers

	FC N (%)	FCF N (%)	Crude OR (95 % CI) *P*-value	Adjusted OR^a^ (95 % CI) *P*-value	Adjusted OR^b^ (95 % CI) *P*-value
	(*N* = 149)	(*N* = 398)			
1 month follow-up					
7-day point prevalence	24 (16.1)	74 (18.6)	1.19 (0.72–1.97) 0.50	1.02 (0.60–1.73) 0.94	1.05 (0.60–1.85) 0.86
3 month follow-up					
7-day point prevalence	26 (17.4)	92 (23.1)	1.42 (0.88–2.31) 0.15	1.30 (0.79–2.14) 0.31	1.35 (0.79–2.31) 0.27
1 month continuous abstinence	23 (15.4)	73 (18.3)	1.23 (0.74–2.05) 0.43	1.11 (0.65–1.88) 0.71	1.17 (0.67–2.05) 0.59
6 month follow-up					
7-day point prevalence	24 (16.1)	103 (25.9)	1.82 (1.11–2.97) 0.02	1.70 (1.03–2.82) 0.04	1.92 (1.12–3.32) 0.02
1 month continuous abstinence	23 (15.4)	95 (23.9)	1.72 (1.04–2.83) 0.03	1.64 (0.98–2.74) 0.06	1.82 (1.05–3.16) 0.03
3 month continuous abstinence	22 (14.8)	77 (19.3)	1.39 (0.83–2.32) 0.22	1.31 (0.77–2.24) 0.32	1.48 (0.83–2.62) 0.19
12 month follow-up					
7-day point prevalence	22 (14.8)	105 (26.4)	2.07 (1.25–3.43) 0.005	2.08 (1.24–3.50) 0.006	2.34 (1.34–4.10) 0.003
6 month continuous abstinence	16 (10.7)	78 (19.6)	2.03 (1.14–3.60) 0.02	2.14 (1.19–3.88) 0.01	2.41 (1.28–4.52) 0.006

Face-to-face counseling plus follow-up telephone counseling, FCF; Face-to-face counseling only, FC; Odds ratio, OR; Confidence Interval, CI

^a^Adjusted for demographic characteristics, perceived difficulty of quitting, willingness to pay for quitting and year of the first visit

^b^Adjusted for all factors in Table 1 (with the exception of cigarette consumption) and year of the first visit

The results on prevalence quit rates were quite similar by complete case analysis (Additional file 1: Table S2).

By intention to treat and complete case analysis, the quit rates at two different time periods (October 2008-December 2010 and January 2012-August 2013) of FCF group at 1, 3, 6 and 12 month follow-up all showed no statistically significant differences (Additional file 1: Table S3), suggesting no period effect.

### Predictors of quitting

Because cigarette consumption was a major item in Fagerström test, it was not included in the stepwise logistic regression model. Table 3 shows that grouping and Fagerström test score were both strong predictors of 7-day point and 6 month continuous quitting at 12 month follow-up.

**Table 3:** By intention to treat, logistic regression (stepwise) analysis for adjusted OR for predictors of quitting at 12 month follow-up

Predictors	Adjusted OR (95 % CI)^a^	*P*-value	P for trend
7-day point prevalence			
Group			
FC (*N* = 149)	1.00		
FCF (*N* = 398)	2.12 (1.26–3.56)	0.005	
Fagerström test score			
Severe (6–10)	1.00		<0.001
Moderate (4–5)	1.79 (1.03–3.10)	0.04	
Low (0–3)	3.02 (1.86–4.90)	<0.001	
Age (years)			
< 31	1.00		0.02
31–40	2.20 (1.16–4.19)	0.02	
41–50	1.32 (0.67–2.62)	0.43	
> 50	2.49 (1.24–5.00)	0.01	
Number of other smokers in household			
0	1.00		
≥ 1	1.81 (1.07–3.05)	0.03	
6 month continuous abstinence			
Group			
FC (*N* = 149)	1.00		
FCF (*N* = 398)	2.17 (1.21–3.90)	0.01	
Fagerström test score			
Severe (6–10)	1.00		<0.001
Moderate (4–5)	2.16 (1.18–3.96)	0.01	
Low (0–3)	3.07 (1.78–5.32)	<0.001	
Marital status			
Single or divorced	1.00		
Married	2.79 (1.15–6.79)	0.02	
Occupation			
Currently employed	1.00		
Student/unemployed/retired/others	1.80 (1.05–3.11)	0.03	

Face-to-face counseling plus follow-up telephone counseling, *FCF* Face-to-face counseling only, *FC* Odds ratio, *OR* Confidence Interval *CI*

^a^Adjusted for all factors in Table 1 (with the exception of cigarette consumption) and year of the first visit

For 6 month continuous abstinence quit rate, Fagerström test score was a strong predictor of quitting with a negative dose–response relationship. Compared to the score of 0–3, the OR (95 % CI) of quitting for Fagerström score of 4–5 and 6–10 was 2.16 (1.18–3.96) and 3.07 (1.78–5.32), respectively. FCF group (2.17, 1.21–3.90), being married (2.79, 1.15–6.79) and unemployed (1.80, 1.05–3.11) were also significant independent predictors of quitting.

Older age and having other smoker(s) in the household were also significant predictors of 7-day point quit rate at 12 month follow-up.

By complete case analysis, the predictors of quitting were similar (Additional file 1: Table S4).

Additional file 1: Table S5 shows that at 3, 6 and 12 month follow-up, the new quit and relapse rates were: 3-month: FC 26.9 % and 19.2 %, FCF 34.8 % and 15.2 %; 6-month: FC 12.5 % and 20.8 %, FCF 25.2 % and 13.6 %; 12-month: FC 13.6 % and 22.7 %, FCF 22.9 % and 21.0 %, respectively. At 12 months, the relapse rates in the FC and FCF groups were similar but the new quit rate in the FCF group was almost two times that in the FCF group, which was not significant, possibly due to small numbers.

## Discussion

Our SCC is one of the longest running part-time regular cessation clinics in China mainland where evidence on the effectiveness of various smoking cessation interventions is scarce. Using systematically collected data from real-world practice, we evaluated the effectiveness of combining one session face-to-face counseling (40 min) plus four telephone follow-up sessions of brief counseling (15–20 min each) as compared to the same face-to-face counseling alone (control group). Our study has provided new evidence of the effectiveness of the additional follow-up telephone sessions and identified predictors of successful quitting in Chinese male smokers. Our experience and findings have demonstrated the feasibility and acceptability of such interventions, and the benefits of developing a ‘model’ smoking cessation clinic as a platform for research and development.

By intention to treat, at 12 month follow-up, the 7-day point prevalence and 6 month continuous quit rate of FC and FCF were 14.8 % and 26.4 %, and 10.7 % and 19.6 %, respectively. Among the 320 million ever smokers in China, only less than 4 % had stopped smoking for at least 2 years [18]. The much higher quit rates in our clinic could be due to the stronger motivation to quit in smokers actively seeking help from a clinic. Our quit rates could serve as references for non-pharmacological interventions in other smoking cessation clinics in China.

Our quit rates, with no medications, appeared to be quite similar to many other RCTs. An RCT in Germany showed that the 7-day point prevalence quit rate of the group without additional follow-up telephone sessions was 16.8 %, and that of the group with additional follow-up telephone sessions was 30.3 % at 6 month follow-up [5]. Zhu et al. reported that the 1 month continuous abstinence quit rate at 6 month follow-up was 14.1 % in 5 sessions with two additional follow-up sessions compared to 7.6 % with self-help booklets alone [7]. In the California Cancer Center, Berndt et al. reported that the 12-month continuous abstinence rates for those who made a quit attempt were 14.7 % for the self-help quit kit group and 26.7 % for the group with the self-help quit kit plus 6 telephone counseling sessions [6].

At 1, 3, 6 and 12 month follow-up, the 7-day point prevalence quit rate of our FC group was relatively stable, but the rate in the FCF group increased steadily. Our 12 month 7-day point prevalence quit rate and 6 month continuous abstinence rate were higher in the FCF than FC group (the adjusted OR and 95 % CI was 2.08, 1.24–3.50 and 2.14, 1.19–3.88, respectively). By complete case analysis, as expected, the differences were greater and more significant, suggesting greater effectiveness of FCF. These, together with the results from comparing new quit rates and relapse rates, indicated that the additional follow-up telephone sessions had some effect on late-onset quitting, mainly by promoting more new quitting and preventing a few relapses. Prospective studies with a larger sample size are needed to examine late-onset quitting and smoking relapse prevention.

We identified that smokers who had lower nicotine dependence, were older, married or unemployed were more likely to quit in the present study. Smokers with lower nicotine dependence had little withdrawal symptoms, and thus they can quit more easily than those smokers with higher nicotine dependences [24–29]. Smokers with advancing age have increased concern about their health status and adverse health consequences of tobacco use, and thus they often have more intention to quit smoking [24, 27–30]. Given that older smokers are often more motivated to quit, the older age of the unemployed group (including the retired) may have contributed to this finding. Married smokers were more likely to have stronger social support to remain abstinent than the unmarried ones [27].

The present study had several limitations. Firstly, an important limitation was selection bias since the smokers were not assigned randomly to the FC and FCF group. We did not do a sample size calculation as the quit rates were unpredictable. But there were no important statistically significant differences of demographic characteristics and tobacco related factors between these two groups, as they sought treatment voluntarily from our SCC. Secondly, because our study lasted for about 6 years, possible time period effect might have affected our comparison, although we had adjusted for the variable of ‘year of the first visit’. Thirdly, although the counselors did not know the aim of the research, social desirability bias might be present. The intervention group received more attention from the counseling, and thus might have been more inclined to falsely report abstinence. Fourthly, there was a large difference in the loss to follow-up rates between the two groups (18.8 % in FC vs. 28.1 % in FCF). One possible explanation was that smokers who received the additional follow-up counseling sessions (FCF group) were required to spend more time than the FC group. Smokers with less motivation to quit smoking were more likely to refuse to receive the additional follow-up counseling because they might consider the additional counseling useless. Additionally, both intention to treat analysis and complete case (per protocol) analysis showed that the quit rates of FCF group were significantly higher than the FC group, which indicated that the large difference in the loss to follow-up rates did not substantially affect the results. Finally, about 65 % of our smokers came from outside of Beijing, and thus it was not convenient for them to return to our clinic for a face-to-face follow-up interview. Of the few (about 9 %) who came back eventually, their exhaled carbon monoxide and saliva cotinine (measured by NicAlert) confirmed the quitting status of over 95 %.

## Conclusion

Our study has shown that the service of one session face-to-face counseling plus four telephone follow-up sessions of brief “booster” counseling for smoking cessation is feasible and acceptable to Chinese smokers. To the best of our knowledge, we have provided the first evidence from a smoking cessation clinic in China or any developing countries that regular additional follow-up telephone problem-oriented counseling session can increase the effectiveness of a face-to-face counseling session alone. With systematic data collection, regular follow-up and rigorous evaluation, the new evidence and knowledge generated by our SCC can serve as a model to guide future smoking cessation service developments in China mainland and other middle- and low-income countries.

## Additional file

Additional file 1: Table S1. Demographic characteristics and tobacco related factors of 407 completed and 140 lost to 12 month follow-up. **Table S2.** By complete case (per protocol) analysis, quit rates of two groups at 1, 3, 6 and 12 month follow-up in 407 male smokers. **Table S3.** By intention to treat and complete case (per protocol) analysis, quit rates of two different time period of FCF group smokers at 1, 3, 6 and 12 month follow-up. **Table S4.** By complete case (per protocol) analysis, logistic regression (stepwise) analysis for adjusted OR for predictors of quitting at 12 month follow-up. **Table S5. 7**-day point prevalence relapse and new quit rate at 3, 6 and 12 month follow-up in two groups. **Figure S1.** Quit rates of two groups at 1, 3, 6 and 12 month follow-up, by intention to treat and complete case (per protocol) analysis. (DOC 320 kb)
