# Supplementary material for: Effectiveness of additional follow-up telephone counseling in a smoking cessation clinic in Beijing and predictors of quitting among Chinese male smokers
Source: BMC Public Health. 2016 Jan 22;16:63. doi: 10.1186/s12889-016-2718-5 (PMC4722719; doi:10.1186/s12889-016-2718-5)
Supplement: Additional file 1: Table S1. — Demographic characteristics and tobacco related factors of 407 completed and 140 lost to 12 month follow-up. Table S2. By complete case (per protocol) analysis, quit rates of two groups at 1, 3, 6 and 12 month follow-up in 407 male smokers. Table S3. By intention to treat and complete case (per protocol) analysis, quit rates of two different time period of FCF group smokers at 1, 3, 6 and 12 month follow-up. Table S4. By complete case (per protocol) analysis, logistic regression (stepwise) analysis for adjusted OR for predictors of quitting at 12 month follow-up. Table S5. 7-day point prevalence relapse and new quit rate at 3, 6 and 12 month follow-up in two groups. Figure S1. Quit rates of two groups at 1, 3, 6 and 12 month follow-up, by intention to treat and complete case (per protocol) analysis. (DOC 320 kb) [file 12889_2016_2718_MOESM1_ESM.doc]

| **Appendix Table 1 Demographic characteristics and tobacco related factors of 407 completed and 140 lost to 12 month follow-up** | | | |
| --- | --- | --- | --- |
|  | Completed (N=407) | Lost follow-up (N=140) | P-value |
| **Demographic characteristics** |  |  |  |
| Age (years) number (%) | N (%) | N (%) |  |
| <31 | 72 (17.7) | 28 (20.0) | 0.32 |
| 31-40 | 138 (33.9) | 36 (25.7) |  |
| 41-50 | 123 (30.2) | 45 (32.1) |  |
| >50 | 74 (18.2) | 31 (22.1) |  |
| Marital status |  |  |  |
| Married | 358 (88.0) | 121 (86.4) | 0.64 |
| Single or divorced | 49 (12.0) | 19 (13.6) |  |
| Education |  |  |  |
| College and above | 247 (60.7) | 78 (55.7) | 0.30 |
| High school and below | 160 (39.3) | 62 (44.3) |  |
| Occupation |  |  |  |
| Currently employed | 325 (79.9) | 111 (79.3) | 0.89 |
| Student/unemployed/retired/others | 82 (20.1) | 29 (20.7) |  |
| Family income per month (Yuan, U.S.$1=6 Yuan) |  |  |  |
| <3000 | 146 (35.9) | 57 (40.7) | 0.29 |
| 3000~6000 | 109 (26.8) | 41 (29.3) |  |
| >6000 | 152 (37.3) | 42 (30.0) |  |
| **Tobacco related factors** |  |  |  |
| Age at initiation of smoking (years) |  |  |  |
| <18 | 139 (34.2) | 44 (31.4) | 0.56 |
| ≥18 | 268 (65.8) | 96 (68.6) |  |
| Cigarettes smoked on average daily (cig/d) |  |  |  |
| ≥20 | 247 (60.7) | 90 (64.3) | 0.48 |
| 10-19 | 117 (28.7) | 40 (28.6) |  |
| <10 | 43 (10.6) | 10 (7.1) |  |
| Smoking duration (years) |  |  |  |
| <20 | 177 (43.5) | 57 (40.7) | 0.57 |
| ≥20 | 230 (56.5) | 83 (59.3) |  |
| Prior attempts to quit smoking |  |  |  |
| 0 | 99 (24.3) | 36 (25.7) | 0.74 |
| ≥1 | 308 (75.6) | 104 (74.3) |  |
| Fagerström test score |  |  |  |
| Severe (6-10) | 178 (43.7) | 68 (48.6) | 0.58 |
| Moderate (4-5) | 100 (24.6) | 33 (23.6) |  |
| Low (0-3) | 129 (31.7) | 39 (27.9) |  |
| Exhaled CO level at first visit (mean:12 ppm) |  |  |  |
| ≥12 | 190 (46.7) | 72 (51.4) | 0.33 |
| <12 | 217 (53.3) | 68 (48.6) |  |
| Stage of quitting smoking |  |  |  |
| Contemplation | 96 (23.6) | 38 (27.1) | 0.43 |
| Preparation | 144 (35.4) | 53 (37.9) |  |
| Action | 167 (41.0) | 49 (35.0) |  |
| Perceived importance of quitting (mean score:86) |  |  |  |
| <86 | 170 (41.8) | 58 (41.4) | 0.94 |
| ≥86 | 237 (58.2) | 82 (58.6) |  |
| Perceived difficulty in quitting (mean score:73) |  |  |  |
| ≥73 | 238 (58.5) | 79 (56.4) | 0.67 |
| <73 | 169 (41.5) | 61 (43.6) |  |
| Perceived confidence in quitting (mean score:68) |  |  |  |
| <68 | 186 (45.7) | 63 (45.0) | 0.89 |
| ≥68 | 221 (54.3) | 77 (55.0) |  |
| Expenditure on cigarettes per day, Yuan (mean:20) |  |  |  |
| <20 | 199 (48.9) | 62 (44.3) | 0.35 |
| ≥20 | 208 (51.1) | 78 (55.7) |  |
| Willingness to pay for quitting, Yuan (mean:2000) |  |  |  |
| <2000 | 221 (54.3) | 66 (47.1) | 0.14 |
| ≥2000 | 186 (45.7) | 74 (52.9) |  |
| Perceived health status at the first visit |  |  |  |
| Fair / poor / very poor | 269 (66.1) | 94 (67.1) | 0.82 |
| Very good / good | 138 (33.9) | 46 (32.9) |  |
| Number of other smokers in household |  |  |  |
| 0 | 331 (81.3) | 115 (82.1) | 0.83 |
| ≥1 | 76 (18.7) | 25 (17.9) |  |
| Medical advice to quit | 139 (34.2) | 48 (34.3) | 0.98 |
| Had doctor diagnosed tobacco related chronic diseases | 218 (53.6) | 72 (51.4) | 0.66 |
| Current drinkers | 282 (69.3) | 98 (70.0) | 0.88 |

| **Appendix Table 2 By complete case (per protocol) analysis, quit rates of two groups at 1, 3, 6 and 12 month follow-up in 407 male smokers** | | | | | |
| --- | --- | --- | --- | --- | --- |
|  | FC N (%) | FCF N (%) | Crude OR (95%CI) P-value | Adjusted OR^1^ (95%CI) P-value | Adjusted OR^2^ (95%CI) P-value |
|  | (N=121) | (N=286) |  |  |  |
| 1 month follow-up |  |  |  |  |  |
| 7-day point prevalence | 24 (19.8) | 74 (25.9) | 1.41 (0.84-2.37) 0.19 | 1.19 (0.69-2.06) 0.52 | 1.09 (0.60-1.97) 0.79 |
| 3 month follow-up |  |  |  |  |  |
| 7-day point prevalence | 26 (21.5) | 92 (32.2) | 1.73 (1.05-2.86) 0.03 | 1.51 (0.90-2.54) 0.12 | 1.47 (0.84-2.58) 0.18 |
| 1 month continuous abstinence | 23 (19.0) | 73 (25.5) | 1.46 (0.86-2.47) 0.16 | 1.27 (0.73-2.18) 0.40 | 1.20 (0.67-2.15) 0.54 |
| 6 month follow-up |  |  |  |  |  |
| 7-day point prevalence | 24 (19.8) | 103 (36.0) | 2.28 (1.37-3.78) 0.002 | 2.07 (1.23-3.49) 0.006 | 2.17 (1.23-3.84) 0.008 |
| 1 month continuous abstinence | 23 (19.0) | 95 (33.2) | 2.12 (1.26-3.55) 0.004 | 1.98 (1.16-3.37) 0.01 | 2.02 (1.13-3.59) 0.02 |
| 3 month continuous abstinence | 22 (18.2) | 77 (26.9) | 1.66 (0.98-2.82) 0.06 | 1.52 (0.87-2.63) 0.14 | 1.58 (0.87-2.87) 0.13 |
| 12 month follow-up |  |  |  |  |  |
| 7-day point prevalence | 22 (18.2) | 105 (36.7) | 2.61 (1.55-4.39) <0.001 | 2.54 (1.49-4.35) 0.001 | 2.75 (1.53-4.94) 0.001 |
| 6 month continuous abstinence | 16 (13.2) | 78 (27.3) | 2.46 (1.37-4.43) 0.003 | 2.53 (1.38-4.64) 0.003 | 2.64 (1.38-5.05) 0.003 |
| Face-to-face counseling plus follow-up telephone counseling, FCF; Face-to-face counseling only, FC; Odds ratio, OR; Confidence Interval, CI.  ^1^ Adjusted for demographic characteristics, perceived difficulty of quitting, willingness to pay for quitting and year of the first visit  ^2^ Adjusted for all factors in table 1 (with the exception of cigarette consumption) and year of the first visit | | | | | |

| **Appendix Table 3 By intention to treat and complete case (per protocol) analysis, quit rates of two different time period of FCF group smokers at 1, 3, 6 and 12 month follow-up** | | | |
| --- | --- | --- | --- |
|  | FCF N (%) (2008.10-2010.12) | FCF N (%) (2012.1-2013.8) | P-value |
| **Intention to treat** | (N=254) | (N=144) |  |
| 1 month follow-up |  |  |  |
| 7-day point prevalence | 47 (18.5) | 27 (18.8) | 0.95 |
| 3 month follow-up |  |  |  |
| 7-day point prevalence | 59 (23.2) | 33 (22.9) | 0.94 |
| 1 month continuous abstinence | 47 (18.5) | 26 (18.1) | 0.91 |
| 6 month follow-up |  |  |  |
| 7-day point prevalence | 65 (25.6) | 38 (26.4) | 0.86 |
| 1 month continuous abstinence | 59 (23.2) | 36 (25.0) | 0.69 |
| 3 month continuous abstinence | 50 (19.7) | 27 (18.8) | 0.82 |
| 12 month follow-up |  |  |  |
| 7-day point prevalence | 71 (28.0) | 34 (23.6) | 0.35 |
| 6 month continuous abstinence | 50 (19.7) | 28 (19.4) | 0.95 |
| **Complete case (per protocol)** | (N=175) | (N=111) |  |
| 1 month follow-up |  |  |  |
| 7-day point prevalence | 47 (26.9) | 27 (24.3) | 0.63 |
| 3 month follow-up |  |  |  |
| 7-day point prevalence | 59 (33.7) | 33 (29.7) | 0.48 |
| 1 month continuous abstinence | 47 (26.9) | 26 (23.4) | 0.52 |
| 6 month follow-up |  |  |  |
| 7-day point prevalence | 65 (37.1) | 38 (34.2) | 0.62 |
| 1 month continuous abstinence | 59 (33.7) | 36 (32.4) | 0.82 |
| 3 month continuous abstinence | 50 (28.6) | 27 (24.3) | 0.43 |
| 12 month follow-up |  |  |  |
| 7-day point prevalence | 71 (40.6) | 34 (30.6) | 0.09 |
| 6 month continuous abstinence | 50 (28.6) | 28 (25.2) | 0.54 |
| Face-to-face counseling plus follow-up telephone counseling, FCF; Face-to-face counseling only, FC. | | | |

| **Appendix Table 4 By complete case (per protocol) analysis, logistic regression (stepwise) analysis for adjusted OR for predictors of quitting at 12 month follow-up** | | | | |
| --- | --- | --- | --- | --- |
| Predictors | Adjusted OR (95%CI)* |  | P-value | P for trend |
| **7-day point prevalence** |  |  |  |  |
| Group |  |  |  |  |
| FC (N=121) | 1.00 |  |  |  |
| FCF (N=286) | 2.50 (1.45-4.32) |  | 0.001 |  |
| Age (years) |  |  |  |  |
| <31 | 1.00 |  |  | 0.03 |
| 31-40 | 2.13 (1.07-4.27) |  | 0.03 |  |
| 41-50 | 1.37 (0.66-2.85) |  | 0.41 |  |
| >50 | 2.70 (1.26-5.76) |  | 0.01 |  |
| Prior attempts to quit smoking |  |  |  |  |
| 0 | 1.00 |  |  |  |
| ≥1 | 1.82 (1.04-3.20) |  | 0.04 |  |
| Fagerström test score |  |  |  |  |
| Severe (6-10) | 1.00 |  |  | <0.001 |
| Moderate (4-5) | 1.74 (0.97-3.12) |  | 0.07 |  |
| Low (0-3) | 2.99 (1.78-5.05) |  | <0.001 |  |
| Number of other smokers in household |  |  |  |  |
| 0 | 1.00 |  |  |  |
| ≥1 | 1.85 (1.04-3.27) |  | 0.04 |  |
| **6 month continuous abstinence** |  |  |  |  |
| Group |  |  |  |  |
| FC (N=121) | 1.00 |  |  |  |
| FCF (N=286) | 2.51 (1.38-4.59) |  | 0.003 |  |
| Marital status |  |  |  |  |
| Single or divorced | 1.00 |  |  |  |
| Married | 2.74 (1.10-6.83) |  | 0.03 |  |
| Occupation |  |  |  |  |
| Currently employed | 1.00 |  |  |  |
| Student/unemployed/retired/others | 1.85 (1.04-3.29) |  | 0.04 |  |
| Fagerström test score |  |  |  |  |
| Severe (6-10) | 1.00 |  |  | 0.001 |
| Moderate (4-5) | 2.16 (1.15-4.05) |  | 0.02 |  |
| Low (0-3) | 2.95 (1.67-5.23) |  | <0.001 |  |
| Face-to-face counseling plus follow-up telephone counseling, FCF; Face-to-face counseling only, FC; Odds ratio, OR; Confidence Interval, CI. | | | | |

| **Appendix Table 5 7-day point prevalence relapse and new quit rate at 3, 6 and 12 month follow-up in two groups** | | | | |
| --- | --- | --- | --- | --- |
|  | FC  (N=149) | FCF  (N=398) | Crude OR (95%CI) | P-value |
| 3 month follow-up | N (%) | N (%) |  |  |
| Relapse / 7-day point prevalence quit | 5/26 (19.2) | 14/92 (15.2) | 0.75 (0.24-2.33) | 0.62 |
| New quit / 7-day point prevalence quit | 7/26 (26.9) | 32/92 (34.8) | 1.45 (0.55-3.81) | 0.45 |
| 6 month follow-up |  |  |  |  |
| Relapse / 7-day point prevalence quit | 5/24 (20.8) | 14/103 (13.6) | 0.59 (0.19-1.86) | 0.37 |
| New quit / 7-day point prevalence quit | 3/24 (12.5) | 26/103 (25.2) | 2.36 (0.65-8.58) | 0.18 |
| 12 month follow-up |  |  |  |  |
| Relapse / 7-day point prevalence quit | 5/22 (22.7) | 22/105 (21.0) | 0.90 (0.30-2.71) | 0.85 |
| New quit / 7-day point prevalence quit | 3/22 (13.6) | 24/105 (22.9) | 1.88 (0.51-6.89) | 0.34 |
| Face-to-face counseling plus follow-up telephone counseling, FCF; Face-to-face counseling only, FC; Odds ratio, OR; Confidence Interval, CI. | | | | |

**
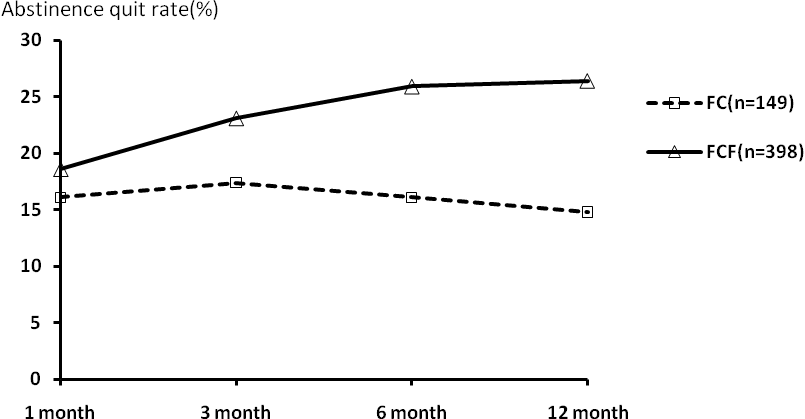
**

**
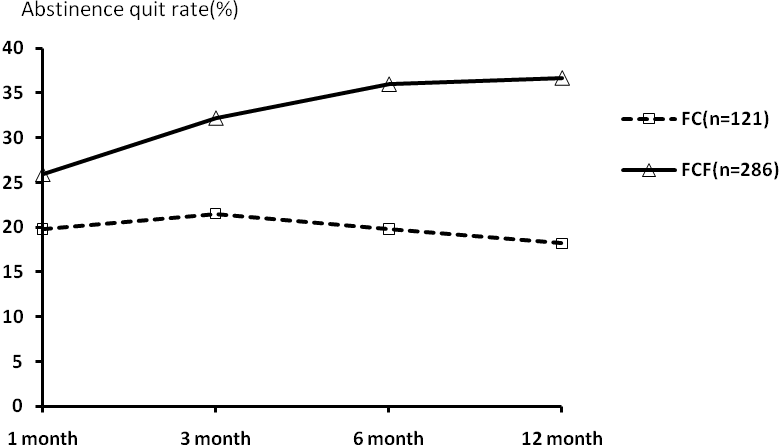
**

**Appendix Figure 1 Quit rates of two groups at 1, 3, 6 and 12 month follow-up, by intention to treat and complete case (per protocol) analysis**

Face-to-face counseling plus follow-up telephone counseling, FCF; Face-to-face counseling only, FC
